# Comparison of a Wearable Tracker with Actigraph for Classifying Physical Activity Intensity and Heart Rate in Children

**DOI:** 10.3390/ijerph16152663

**Published:** 2019-07-25

**Authors:** Seoungki Kang, Youngdeok Kim, Wonwoo Byun, JinSu Suk, Jung-Min Lee

**Affiliations:** 1 Graduate School of Education, Yongin Univerisity, 134 Yongindaehak-ro, cheoin-gu, Yongin-si 449-714, Korea; 2 Department of Kinesiology and Health Sciences, Virginia Commonwealth University, Richmond, VA 23284, USA; 3 Department of Health, Kinesiology, and Recreation, University of Utah, 250 S 1850 E (HPER North), Room 205, Salt Lake City, UT 84108, USA; 4 Department of Physical Education, College of Physical Education, Kyung Hee University, 1732 Deogyeong-daero, Giheung-Gu, Yongin-si 17104, Korea

**Keywords:** accelerometer, Fitbit, physical activity classification

## Abstract

*Introduction:* To examine the validity and reliability of the Fitbit Charge HR (FCH), wrist-worn ActiGraph (AG) accelerometers were used for assessing the classification of physical activity (PA) into intensity categories in children. *Methods:* Forty-three children (n = 43) participated in the study. Each participant completed 3 min bouts of 12 PAs ranging from sedentary to vigorous intensity while simultaneously wearing FCH and AG on both hands, a Polar HR monitor, and a portable indirect calorimeter. Total time spent in different PA intensity levels measured by FCH and AG were compared to the indirect calorimetry. *Results:* The highest classification accuracy values of sedentary behavior was 81.1% for FCH. The highest classification (72.4%) of light intensity PA was observed with Crouter’s algorithm from the non-dominant wrist. Crouter’s algorithm also show the highest classification (81.8%) for assessing moderate to vigorous intensity PA compared to FCH (70.8%). Across the devices, a high degree of reliability was found in step measurements, ranging from an intra-class correlation (ICC) = 0.92 to an ICC = 0.94. The reliability of the AG and the FCH showed high agreement for each variable. *Conclusion:* The FCH shows better validity for estimating sedentary behavior and similar validity for assessing moderate to vigorous PA compared to the research-grade monitor. Across the devices, the reliability showed the strongest association.

## 1. Introduction

An accurate assessment of children’s physical activity (PA) is important to identify and quantify PA patterns of children to reverse the childhood obesity epidemic [1]. However, it is challenging to accurately assess children’s PA in a free-living environment because of the recall bias and their intermittent/sporadic activity patterns [2]. In addition, there is still a lack of consensus on the accuracy of a research version of activity monitors (i.e., ActiGraph) for use in PA and epidemiological research in children. Several accelerometry-based cut points have been introduced to accurately classify intensity of activities in children [3,4]. However, there is currently no established consensus on which cut-points should be employed nor where the monitor should be placed (i.e., waist vs. wrist). In addition, accelerometry-based activity monitors do not adequately capture some specific types of movements such as weight-bearing activities (i.e., weight lifting), stationary (i.e., cycling) or gliding activities (i.e., skating), and only involved upper body movement [5].

The ActiGraph (ActiGraph LLC, Pensacola, FL, USA) is one of the most commonly utilized accelerometers for assessing children’s PA in free-living conditions [6]. However, it is expensive (around $250 per device) and purchasing the ActiGraph software license necessary to process, manage, and analyze its data involves additional expenses (around $1700). On the other hand, the Fitbit Charge HR (Fitbit Inc., San Francisco, CA, USA) is a relatively new accelerometry-based activity tracker that has been developed for consumers. The Fitbit brand is one of the popular wearable activity trackers and the price of the tracker is around $100 and users are able to easily track their activity through its LED display and an application on their smartphone. Furthermore, minute-by-minute PA data can be downloaded from the third-party website (i.e., Fitabase.com) for researchers and practitioners with the subscription-based fee. Fitbit brand trackers are not only widely used in research and clinical settings for assessing individuals’ PA but also utilized as intervention tools to promote physical activity.

Both the research and consumer version of activity monitors can be worn on the wrist to improve participant/user compliance in terms of wear time and wear position [7]. Previously conducted studies indicated that the Fitbit Flex showed moderate validity for measuring PA relative to direct observation and the ActiGraph in adults [8,9]. Fitbit One trackers appear to accurately and reliably measure step counts in healthy young populations during treadmill walking [10]. One of the studies indicated that waist-oriented wearable trackers (i.e., Fitbit Zip and Fitbit One) revealed most accurate measures of step count in three different conditions (i.e., treadmill, over-ground, and free-living condition) in adults [11]. Several research studies have examined the validity of Fitbit monitors in adults [12,13,14,15,16]. However, to the best of our knowledge, none of the studies investigated the feasibility for the consumer wearable tracker (i.e., Fitbit Charge HR) in terms of activity intensity classification and heart rate measure in children. Therefore, the primary purpose of the study was to examine the validity and reliability of the Fitbit Charge HR and two previously developed children’s cut points (Chandler’s and Crouter’s) utilized for wrist-worn Actigraph monitors against the criterion measures (i.e., indirect calorimetry) on activity classification. The secondary purpose of the study was to examine the validity of heart rate measures from the Fitbit Charge HR compared to the Polar heart rate monitor in structured settings.

## 2. Methods

### 2.1. Participants

A total of 43 children (girls = 18 and boys = 25), aged 8–12 years, volunteered to participate in the study and were recruited from adjacent communities of Yong-In, South Korea. Prior to the data collection, each participant and their parent completed an assent and a written informed consent form, and were provided with details on the study protocol before providing consent and assent. Participants were eligible for this study only if they fell within the appropriate age range and had no apparent contraindications to the activity protocol. The study protocols were approved by the institutional review board of Yong-In University.

### 2.2. Procedures

Participants’ standing height and weight were measured to the nearest 0.1 cm/kg. Body mass index (BMI) was calculated and expressed as a percentile based on the population mean BMI values reported in the CDC growth charts [17]. Body composition (i.e., body fat percentage) was measured using dual-energy X-ray absorptiometry (Lunar DXA, General Electric, Boston, MA, USA), following standard procedures [18].

Following the anthropometric measures, each participant was asked to lay down in bed for 10 min for resting and then fitted with a flexible pediatric mask for the assessment of resting energy expenditure (REE) for an additional 10 min and the REE was expressed as mL·kg·min^−1^. Two Fitbit Charge HR trackers and two ActiGraph GT3X+ were worn on the dominant and non-dominant wrist. We randomly counterbalanced the wear position between the ActiGraph and Fitbit tracker on the wrist. The manufacturer’s suggested guidelines were carefully followed for each device. All instruments were synchronized and initialized using the participant’s personal information (i.e., age, gender, height, weight, and handedness) before the measurements. The test was performed at various times of day. However, participants were asked to abstain from eating and exercise for 4 h before the test. Each participant then performed an activity routine that included a series of different activities and lasted 48 min in a gym.

Participants performed each activity for 3 min and there was a 1 min rest between each activity to facilitate transitions and tracking of data. Due to an initialization delay for the monitor, data during the first minute of activity were not selected for the data analysis. Oxygen consumption and heart rate were simultaneously measured throughout the routine with the COSMED K4B^2^ metabolic analyzer and the Polar heart rate monitor. A total of 12 activities that were selected to mimic children’s usual free-living activities were categorized into four distinct PA intensities: (1) sedentary (sitting quietly in a chair, playing a video game, and watching TV), (2) moderate intensity (treadmill walking at 2 and 3 mph, stationary cycling at 80 watts, sweeping, hand weight exercise, and cool-down walking), and (3) vigorous intensity (treadmill running at 5 mph and stationary cycling at 120 watts) (Table 1).

### 2.3. Criterion Measure

The K4b^2^ (COSMED, Rome, Italy) is a portable indirect calorimeter that allows the measurement of oxygen consumption under free-living conditions and it was used as a criterion measurement to examine the intensity of PA in this study. The K4b^2^ has been extensively validated and utilized for examining the physical activity level [19,20]. Before each trial, gas calibrations (16% O_2_, 5% CO_2_) and flow-volume were calibrated with a flow range capacity up to 20 L·s^−1^. A room air calibration and delay calibration were also performed using the manufacturer’s user manual. Breath-by-breath measures of pulmonary ventilation and gas exchange were used to calculate oxygen uptake VO_2_ (mL·kg·min^−1^) and metabolic equivalent tasks (METs) values. METs were computed by dividing the activity VO_2_ by the measured participant’s resting metabolic rate. The Polar RS400 heart rate monitor (Polar Electro, Inc, Lake Success, NY, USA) is also used as a criterion measure for assessing heart rate. The Polar heart rate monitor has been validated with ECG in children [21].

### 2.4. Activity Monitors

The ActiGraph GT3X+ (Pensacola, FL, USA) is a research-grade monitor, the most commonly used accelerometer to assess physical activity in free-living environments. It features a tri-axial accelerometer that records acceleration ranging from 0.05 g to 6.00 g and provide physical activity frequency, intensity, and duration. The ActiGraph accelerometer has been utilized in a subsample of the National Health and Nutrition Examination Survey (NHANES) to provide objective measures of physical activity [22].

The Fitbit Charge HR (Fitbit, San Francisco, CA, USA) is a wrist-worn activity monitor that continuously measures movement and heart rate, using a tri-axial accelerometer and LED light sensor. The Fitbit Charge HR utilizes optical blood flow sensing using photoplethysmography (PPG) techniques to measure heart rate (HR). PPG is a non-invasive method for the detection of HR and is connected with the optical properties of vascular tissue using a probe, usually LEDs. PPG sensors use the probe (e.g., LED light) to shine directly into the skin and interact with changes in the blood volume to configure an HR. The monitor uses these measures to give the wearer information regarding heart rate, PA intensity, energy expenditure (EE), step count, distance traveled, and stairs climbed (increasing 10 ft based on atmospheric pressure). The Fitbit Charge HR uses its proprietary algorithm; the Fitbit Charge HR transforms acceleration signals into activity counts in 60 s sampling intervals that define PA intensities as 0 = sedentary, 1 = light PA, 2 = moderate PA, and 3 = vigorous PA. The Fitbit Charge HR has a small screen on the band so the wearer can track their progress. The band can also be connected to a mobile phone app or synced to a PC to track patterns over time.

### 2.5. Data Processing

Breath-by-breath data from the indirect calorimetry were aggregated to provide average minute-by-minute data to facilitate integration with the activity intensity classification from each monitor. The last 5 min average values of the resting metabolic rate were used as one metabolic equivalent of task (i.e., 1 MET) to categorize children’s physical activity intensity (measured-METs). The raw ActiGraph accelerometer data for each axis and the mean vector magnitude (VM: The square root of the sum of squares of each of the three axes) were converted to counts per 5 s, then collapsed into minute-by-minute data in order for the comparisons. Fitbits’ minute-by-minute data (i.e., PA intensity, energy expenditure, and HR) was downloaded from the Fitabase website (Small Steps Labs LLC, San Diego, CA, USA). All methods were compared across the measured METs for PA (sedentary, light PA, and moderate and vigorous PA (MVPA)). Crouter’s and Chandler’s wrist cut-points [3,4] were applied to the data to create a dichotomous categorization for every minute of the protocol to the measured METs, and Polar heart rate data was downloaded and aggregated to provide average minute-by-minute data to examine the accuracy of the measured HR from the Fitbit Charge HR. Following Welk’s recommendation for designing accelerometer-based value calibration, all activities were performed progressively from sedentary to vigorous intensity [23]. Data during the first minute of each activity was removed for data analysis due to the time delay in attaining a steady-state condition at the start and end of each activity [24].

### 2.6. Statistical Analyses

Descriptive statistics were calculated to summarize the demographic information for the participants. Overall agreement and classification accuracy of the Fitbit Charge HR against measured child-METs were evaluated using the following statistical analyses: (1) Cohen’s kappa evaluating the levels of agreement on activity intensity classification between the three methods and measured METs [25], (2) sensitivity (Se), specificity (Sp), and area under the receiver operating curve (ROC-AUC) to determine the classification accuracy of the three methods with/without cycling activities, (3) mean absolute percentage error (MAPE) was calculated to find the overall measurement error for the heart rate comparison, and a repeated measures ANOVA with Bonferroni post-hoc corrections were used to test for differences among the three HR measures, and (4) intra-class correlation (ICC) estimates and their 95% confidence intervals were calculated to examine the reliability for Fitbit and two ActiGraph cut-points on each variable (i.e., MVPA, energy expenditure, steps, HR, and vector magnitude) (dominant vs. non-dominant) based on a mean-rating (k = 2), absolute-agreement, and 2-way mixed-effects model suggested by Koo et al. [26]. Cronbach’s Alpha was used to measure the strength of the consistency, and Friedman’s Chi-square was calculated to test differences between groups. In addition, normality was tested by the Kolmogorov–Smirnov test and the Shaprio–Wilk test. All statistical analyses were performed using STATA Version 14 (StataCorp, College Station, TX, USA), and statistical significance was set at α = 0.05.

## 3. Results

Table 2 presents the descriptive statistics for demographic characteristics of children (n = 43). The mean ± SD was calculated to be 9.7 ± 1.3 years for age, 144.5 ± 9.6 cm for height, 37.8 ± 8.1 kg for weight, 17.9 ± 2.2 m·kg^−2^ for BMI, 57.9 ± 25.1% for BMI percentile, resting heart rate 105.6 beats·min^−1^, and 19.4 ± 6.6% for body fat. Only two children reported their left hand is their dominant hand.

Table 3-1 summarizes statistics for classification accuracy of six different methods. When compared with the criterion measure (i.e., indirect calorimetry), on average, the Fitbit Charge HR revealed the highest classification accuracy (80.73%), high sensitivity (91.6%), moderate specificity (72.4%), and a high ROC-AUC value (0.82) for sedentary behavior. The statistics for the Fitbit Charge HR were better than those for the other methods for sedentary behavior. For the light PA classification, on average, the Crouter’s cut-points worn on the right showed the highest classification accuracy (72.42%), low sensitivity (26.4%), high specificity (94.0%), and a moderate ROC-AUC value (0.61). The overall statistical agreement for the light activity classification showed better with both research-grade monitors.

Table 3-2 presents the agreements in MVPA classification with/without cycling activity. The sensitivity, percentage of correctly classified intensity, kappa coefficients, and ROC-AUC values yield better agreement without cycling activity. The highest MVPA classification (90.1%), sensitivity (74.6%), and ROC-AUC value (.60) were observed with Crouter’s cut-points worn on the right without cycling. However, Chandler’s cut-points showed the highest specificity (100%) but revealed the lowest sensitivity (14.8%). Overall, the sensitivity improved in general ranging from 2.79% to 19.93%, the percentage of correctly classified intensity improved ranging from 5.85% to 9.46%, and kappa coefficients increased ranging from 0.06 to 0.46. The ROC-AUC values also increased, ranging from 0.05 to 0.09 in the devices. However, the percentage of specificity remained at the same level regardless of the methods.

The results from the intra-class correlation coefficient (ICC) are illustrated in Table 4. Across the devices, a high degree of reliability was found in step measurements ranging from ICC = 0.92 to ICC = 0.94. The reliability of the Fitbit Charge HR was good in general (ICC = 0.75–ICC = 0.94) on each variable. Chandler’s MVPA classification showed poor ICC = 0.278.

**Figure 1:**
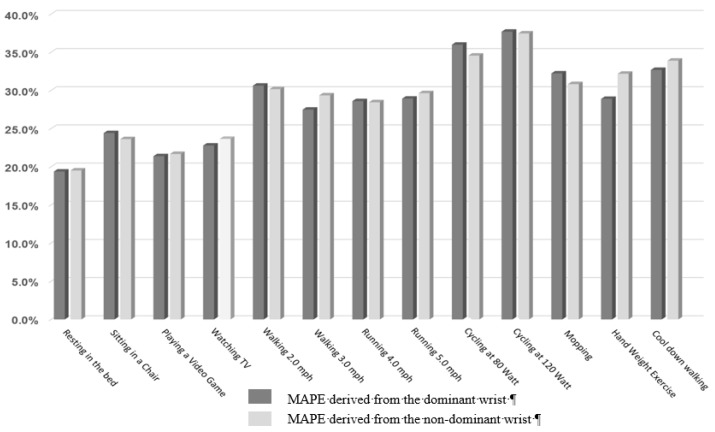
Mean absolute percentage error for heart rates from the Fitbit Charge trackers (dominant vs. non-dominant).

Figure 1 illustrates the MAPE on heart rates measured by the Fitbit Charge HR. A repeated measures ANOVA revealed that there was a significant difference between the heart rate measured by the Polar heart rate monitor (criterion measure) and those measured by Fitbit Charge HR for non-dominant- and dominant-oriented (F(1.250, 1770.88) = 472.85, *p* = 0.001). However, post-hoc tests using the Bonferroni correction indicated that no significant difference was found between Fitbit Charge HR placed on the non-dominant and the dominant wrist. The average MAPE values of heart rate were 27.31% for the non-dominant-placed tracker and 27.50% for the dominant-placed tracker.

## 4. Discussion

This study examined the validity and reliability of the Fitbit Charge HR tracker and two ActiGraph wrist cut points for classifying PA intensity in 9- to 12-year-old children against indirect calorimetry. The Fitbit Charge HR tracker accurately estimated sedentary activities (i.e., 91.61 (se) and 72.42 (sp)) and the ActiGraph wrist cut points outperformed the FCH in classifying MVPA. In general, good reliability was observed in these trackers regardless of the wear position (i.e., non-dominant vs. dominant). The results from this study demonstrated that the Fitbit Charge HR tracker has comparable validity compared to the indirect calorimetry. Across the intensity classification, the Fitbit Charge HR tracker tended to have high levels of agreements in sedentary activity classification (80.32%) among the methods. The moderate level of agreements in MVPA (70.8%) is relative to the research grade monitor (81.8%). Further, the Fitbit Charge HR did not perform well (i.e., average MAPE = 27.0%) in detecting heart rate in children.

To our knowledge, this is the first study to validate the Fitbit Charge HR for PA intensity classification and compare its accuracy with the two recently published sets of ActiGraph wrist cut-points [3,4]. One study [16] has validated the Fitbit Charge HR tracker in children with congenital heart disease but they examined the accuracy of PA classification based on a step measure against the waist-oriented ActiGraph. They found that the Fitbit Charge HR reported higher step counts than the ActiGraph monitor and device agreement for MVPA was only good for boys, but poorer for the overall participants. Another study [27] has validated the waist-oriented Fitbit One device among children, finding that the Fitbit One step counts showed comparable estimates of habitual physical activity in sedentary and light PA intensity compared to the step-based physical activity intensity classification: Sedentary (0–100 step counts per minute), light (101–2295 step counts per minute), moderate (2296–4011 step counts per minute), and vigorous (over 4012 step counts per minute) activity. The results of the study also showed that strong differences were found in high-intensity activity and weak differences were found in light-intensity activity.

The Fitbit brand trackers have been largely used in intervention studies as a self-monitoring tool for the promotion of physical activity in children [28,29,30,31,32,33]. However, given that the accuracy of the Fitbit monitors for children was unknown, a caveat of these previous studies was that the researchers applied a research-grade monitor such as ActiGraph or a SenseWear armband to ensure a collection of accurate estimates of PA data [28]. However, using those research-grade activity monitors in addition to Fitbit monitors may have hindered achieving high adherence rates due to the increased burden of participants, especially in children. This study shows acceptably reasonable levels of accuracy of the Fitbit monitor (as compared with the Actigraph), suggesting that the Fitbit Charge HR tracker is a viable alternative assessment method for objectively evaluating children’s sedentary behavior in intervention and epidemiological research.

There are several interesting findings in this study. Firstly, the Fitbit Charge HR did not perform well detecting heart rate compared to the Polar heart rate monitor. The findings contrast with a previous study [34] that compared heart rate values with measurements recorded during continuous electrocardiographic (cECG) monitoring in children (8.21 ± 3.09 years) undergoing surgery. In the study, the Fitbit Charge HR-derived HR showed excellent accuracy compared to HRs measured by cECG and Pulse Oximetry (SpO2R) during pediatric surgical procedures. Another study performed by Kroll et al. [35] indicated that the Fitbit Charge HR tended to underestimate heart rate values when heart rate values were in the range of 75 to 120 beats per min. Therefore, more testing of these PPG sensor-derived HR in free-living settings are needed to provide objective evidence in terms of the validity of HR monitoring capabilities in children.

Secondly, this study examined the classification accuracy with/without the cycling activities because accelerometry-based activity monitors have proven very difficult to measure cycling and weight-bearing activity [36]. Given that the actual algorithms used in the Fitbit Charge HR tracker are unknown due to the proprietary rights, it is not clear what types of accelerometer data and/or demographic variables are used in producing physical activity parameters. However, we speculated that the Fitbit tracker might utilize both heart rate and accelerometer information in its algorithms alike the Actiheart device (CamNtech Ltd., Cambridge, UK) which integrates both the heart rate and accelerometer to improve the accuracy of PA measurement. However, the results are consistent with previous research [37,38,39] showing overall improvements in classification accuracy across the monitors without cycling activity (i.e., 6% improvement for Fitbit Charge HR). In this regard, we also quantified the estimate of heart rate values from the Fitbit Charge HR tracker and the overall MAPE was 27.3%. These findings suggest that the Fitbit Charge HR tracker may not integrate the heart rate but the accelerometer information in the algorithms. The estimates of the heart rate from the wrist heart rate sensor may provide a significant advantage over the activity monitors that utilized only accelerometer data. Additional studies should be performed to examine this issue, and subsequent algorithms should take into account heart rate information in order to further improve the accuracy of assessing physical activity patterns because most wearable activity trackers currently available in the market have a built-in wrist heart rate sensor.

Lastly, overall good reliability was observed between the non-dominant and dominant wrist-placed devices and the variables (i.e., MVPA, EE, steps, and heart rate) tested in this study. While most of the devices had moderate reliability between the devices. Only Chandler’s MVPA cut points showed poor reliability (ICC = 0.278) due to the low sensitivity observed in light and MVPA intensity ranging from14.5% to 39.5% which majorly influence the reliability of Chandler’s cut point. The poor reliability may also derive from the difference criterion and methodology used in Chandler’s calibration study. In contrast to this study, they used the heart rate and direct observation as a criterion measure in the gym setting and regression analyses were utilized to develop prediction equations to predict the percentage of heart rate reserve (HRR) from activity counts produced by ActiGraph.

There are several notable strengths of this study. First of all, this study is the first to examine the validity and the reliability of a consumer-grade activity tracker (i.e., Fitbit Charge HR) and a widely utilized research-grade activity monitor (i.e., Actigraph) against indirect calorimetry in children. Moreover, we included a series of activities that simulate children’s free-living activities. In addition, this study is the first of its kind providing evidence on a Fitbit device’s heart rate function in children. However, a few limitations of the study must also be mentioned. The sample population was only healthy children, with a normal range of body weight and body fat between 9 and 12 years old. Findings of this study may not be generalizable to the broader populations of children. One of the challenges to make a direct comparison of the wearable tracker is the lack of transparency regarding the specific algorithms. In addition, there are logistical and ethical challenges to access individual’s user profiles directly for research, and fees involved using the Fitabase website. In addition, the criterion measure for heart rate assessment was the Polar heart rate monitor, instead of ECG.

In conclusion, our findings suggest that the Fitbit Charge HR has similar validity for estimating physical activity intensity in sedentary behavior and shows comparable MVPA intensity estimation compared to the research-grade monitor. Across the devices, reliability was strong between the dominant- and non-dominant-placed monitors. The Fitbit Charge HR provides a favorable outcome for the measurement of heart rate in children by utilizing the built-in HR sensor.

## Figures and Tables

**Table 1:** Description of performed activities by intensity.

Intensity Type	Activity	Duration	Measured METs	Description
Sedentary Activity	Supine position	20 min	1.0 (5.4 mL/kg/min)	Children lay in the supine position on a bed
	Sitting on a chair	3 min	1.4 (7.6 mL/kg/min)	Sitting on a chair quietly
	Playing a Video game	3 min	1.4 (7.6 mL/kg/min)	While sitting on a chair desk, playing a video game provided by a researcher
	Watching TV	3 min	1.3 (7.3 mL/kg/min)	Watching a TV show/movie selected by a researcher while seating on a chair at a desk
Free-living Activity	Mopping	3 min	2.8 (15.0 mL/kg/min)	Mopping a floor on their own pace
	Hand weight exercise	3 min	2.1 (11.5 mL/kg/min)	Lifting a 5 kg (decided by a researcher’s visual inspection on a participant’s physical maturation) dumbbell up and down constantly
Cycling	Stationary cycling at 80 watts	3 min	6.1 (36.8 mL/kg/min)	Cycling a stationary bicycle ergometer at 80 watts
	Stationary cycling at 120 watts	3 min	6.8 (40.9 mL/kg/min)	Cycling a stationary bicycle ergometer at 120 watts
Locomotor Activity	Treadmill walking 2.0 mph	3 min	2.7 (15.8 mL/kg/min)	Walking at 2.0 mph on the treadmill
	Treadmill walking 3.0 mph	3 min	3.5 (20.8 mL/kg/min)	Walking/running at 3.0 mph on the treadmill
	Treadmill running 4.0 mph	3 min	4.7 (29.6 mL/kg/min)	Running at 4.0 mph on the treadmill
	Treadmill running 5.0 mph	3 min	6.0 (37.9 mL/kg/min)	Running at 5.0 mph on the treadmill
	Cool down walking	3 min	2.3 (12.3 mL/kg/min)	Self-paced cool down walking on the treadmill

MET, metabolic equivalent of task.

**Table 2:** Participant’s characteristics (mean ± standard deviation).

Variables	Boys (n = 25)	Girls (n = 18)	Total (n = 43)
Age (years)	9.8 ± 1.3	9.6 ± 1.2	9.7 ± 1.3
Height (cm)	145.9 ± 9.3	142.6 ± 9.9	144.5 ± 9.6
Weight (kg)	39.8 ± 7.6	35.0 ± 8.2	37.8 ± 8.1
BMI (m·kg^−2^)	18.6 ± 2.2	17.0 ± 1.9	17.9 ± 2.2
BMI Percentile (%)	66.8 ± 24.5	45.4 ± 20.6	57.9 ± 25.1
Normal Weight (BMI %)	55.1 ± 21 (n = 17)	43 ± 18.3 (n = 17)	49 ± 20.3 (n = 34)
Overweight (BMI %)	90.3 ± 2.7 (n = 6)	87 (n = 1)	89.9 ± 2.7 (n = 7)
Obese (BMI %)	96 ± 1.4 (n = 2)	N/A	96 ± 1.4 (n = 2)
Resting Heart Rate (beat·min^−1^)	112.5 ± 41.8	99.2 ± 38.6	105.6 ± 40.3
Body Fat (%)	19.8 ± 7.3	18.9 ± 5.7	19.4 ± 6.6

* BMI, Body Mass Index.

**Table 3:** (1) Agreements in SB and Light PA intensity classifications for four methods, Kappa, ROC-AUC, Sensitivity, and Specificity; (2) Agreements in MVPA intensity classifications for four methods, Kappa, ROC-AUC, Sensitivity, and Specificity with/without cycling activity.

Intensity	Methods	Correctly Classified (%)	Sensitivity (%)	Specificity (%)	ROC-AUC (95% CI)	kappa
**(1)**						
**SB**	Fitbit dominant	80.32	91.61	72.42	0.82 (0.80–0.84)	0.61
	Fitbit non-dominant	81.14	90.74	72.7	0.82 (0.80–0.84)	0.60
	Chandler dominant	69.61	100	48.32	0.74 (0.71–0.76)	0.43
	Chandler non-dominant	65.16	96.23	43.41	0.70 (0.67–0.72)	0.35
	Crouter dominant	72.28	100	52.88	0.76 (0.74–0.78)	0.48
	Crouter non-dominant	71.51	100	51.56	0.76 (0.73–0.78)	0.46
**Light PA**	Fitbit dominant	66.1	33.04	81.6	0.57 (0.55–0.59)	0.23
	Fitbit non-dominant	65.98	35.02	80.5	0.58 (0.55–0.59)	0.28
	Chandler dominant	71.99	39.48	87.23	0.63 (0.61–0.65)	0.15
	Chandler non-dominant	67.56	33.66	83.46	0.58 (0.56–0.60)	0.06
	Crouter dominant	69.77	21.41	92.46	0.62 (0.58–0.62)	0.18
	Crouter non-dominant	72.42	26.36	94.02	0.60 (0.58–0.62)	0.17
**(2)**						
**MVPA**	Fitbit Right	70.80	44.62	82.82	0.64 (0.61–0.66)	0.29
	Without Cycling	76.65	54.71	82.89	0.69 (0.67–0.71)	*0.40*
	Fitbit Left	70.80	41.03	84.47	0.63 (0.60–0.65)	0.27
	Without Cycling	77.69	53.62	84.54	0.69 (0.66–0.71)	*0.40*
	Chandler Right	77.43	28.25	100	0.64 (0.62–0.67)	0.35
	Without Cycling	86.60	34.49	100	0.70 (0.67–0.72)	*0.40*
	Chandler Left	70.24	14.57	95.78	0.55 (0.53–0.57)	0.13
	Without Cycling	79.70	22.83	95.88	0.59 (0.56–0.62)	*0.60*
	Crouter Right	81.88	54.71	94.34	0.75 (0.72–0.77)	0.53
	Without Cycling	90.05	74.64	94.43	0.84 (0.82–0.86)	*0.60*
	Crouter Left	77.79	45.74	92.49	0.69 (0.66–0.71)	0.43
	Without Cycling	85.96	62.68	92.58	0.78 (0.75–0.80)	*0.60*

SB, sedentary behavior (<1.5 METs); LPA, light physical activity (1.5–2.99 METs); MVPA, moderate and vigorous physical activity (>3 METs); CI, confidence interval; k, kappa statistic; ROC, receiver operating curve; AUC, area under curve.

**Table 4:** ICC for dominant vs. non-dominant worn trackers using single-rating, absolute-agreement, and 2-way mixed effects models.

Device	Single Measure (Variable)	Intra-class Correlation	95% Confidence Interval				
			Lower Bound	Upper Bound	Cronbach’s Alpha	Friedeman’s	Sig
						Chi-Square	
Fitbit	(MVPA)	0.773	0.751	0.793	0.872	8.533	0.003
	(EE)	0.834	0.817	0.850	0.910	17.088	0
	(Steps)	0.944	0.938	0.950	0.971	0.598	0.439
	(Heart Rate)	0.754	0.731	0.776	0.86	0.027	0.869
ActiGraph	(Crouter MVPA)	0.734	0.71	0.758	0.847	3.967	0.046
	(Chandler MVPA)	0.278	0.229	0.325	0.435	2.597	0.107
	(Steps)	0.919	0.911	0.927	0.958	7.668	0.006
	Vector Magnitude	0.493	0.453	0.532	0.661	2.947	0.086

ICC, intra-class correlation; MVPA, moderate and vigorous physical activity; EE, energy expenditure.
